# Detecting Endotracheal Tube and Carina on Portable Supine Chest Radiographs Using One-Stage Detector with a Coarse-to-Fine Attention

**DOI:** 10.3390/diagnostics12081913

**Published:** 2022-08-07

**Authors:** Liang-Kai Mao, Min-Hsin Huang, Chao-Han Lai, Yung-Nien Sun, Chi-Yeh Chen

**Affiliations:** 1 Department of Computer Science and Information Engineering, National Cheng Kung University, Tainan 701401, Taiwan; 2 Department of Surgery, National Cheng Kung University Hospital, College of Medicine, National Cheng Kung University, Tainan 704302, Taiwan

**Keywords:** endotracheal intubation, object detection, coarse-to-fine attention, deep learning

## Abstract

In intensive care units (ICUs), after endotracheal intubation, the position of the endotracheal tube (ETT) should be checked to avoid complications. The malposition can be detected by the distance between the ETT tip and the Carina (ETT–Carina distance). However, it struggles with a limited performance for two major problems, i.e., occlusion by external machine, and the posture and machine of taking chest radiographs. While previous studies addressed these problems, they always suffered from the requirements of manual intervention. Therefore, the purpose of this paper is to locate the ETT tip and the Carina more accurately for detecting the malposition without manual intervention. The proposed architecture is composed of FCOS: Fully Convolutional One-Stage Object Detection, an attention mechanism named Coarse-to-Fine Attention (CTFA), and a segmentation branch. Moreover, a post-process algorithm is adopted to select the final location of the ETT tip and the Carina. Three metrics were used to evaluate the performance of the proposed method. With the dataset provided by National Cheng Kung University Hospital, the accuracy of the malposition detected by the proposed method achieves 88.82% and the ETT–Carina distance errors are less than 5.333±6.240 mm.

## 1. Introduction

In intensive care units (ICUs), endotracheal intubation is a common medical procedure when a patient cannot spontaneously breathe. When intubating, the position of an endotracheal tube (ETT) needs to be taken into account because some complications can be caused by its malposition. A deep position can cause tachycardia, hypertension, and/or bronchospasm. On the other hand, a shallow position can cause inadvertent extubation or larynx damage [1]. Extensive research has shown that the appropriate depth of an ETT can be determined by the distance between the ETT and Carina [2]. Therefore, to reduce the likelihood of serious complications, it is essential to develop a method to accurately detect the distance between ETT and Carina.

Recent evidence [3] suggests that chest radiography should be performed after endotracheal intubation. However, in ICUs, chest radiographs (CXRs) are usually obtained in supine anteroposterior (AP) view by a portable X-ray machine. In this situation, several challenges limit the detection accuracy; for example, external monitoring devices, tubes, or catheters can cause ambiguity in the position of an ETT and Carina. Additionally, the image quality of CXR obtained in the supine AP view is lower than the standard posteroanterior (PA) view. Therefore, over the past decade, researchers pay increased attention to computer-aided detection (CAD) methods in an attempt to improve the detection of unsuitable endotracheal intubation and reduce the burden on doctors.

Previous automatic CAD methods for ETTs detection [4,5,6] have a similar procedure, which can be simplified into four steps, i.e., preprocessing, finding neck, seed generation, and region growing. These methods use feature extraction to detect the ETT and have achieved acceptable results. However, such approaches have failed to address the problem that the manual templates and hyperparameters should be determined based on experience or morphology in the procedure. These shortages may limit the ability of generalization and increase the responsibility of researchers.

During the same period, the success of deep convolutional neural networks (CNN) in image classification catch researchers’ attention. Therefore, some studies apply CNN to detect the malposition, trying to simplify the feature extraction process. Lakhani [7] used a CNN-based classifier to identify the presence or absence and the low or normal positioning of the ETT. Frid-Adar et al. [8] used the physical properties of ETTs and a public dataset of chest radiographs to synthesize an ETT over real X-ray images. After that, they used a U-Net shaped CNN to detect and segment ETTs. Lakhani et al. [9] used Inception V3 [10] to classify CXRs into 12 categories with the distance between ETT tip and Carina, including bronchial insertion, the distance range from 0 cm to 10 cm (0.0–0.9 cm, 1.0–1.9 cm, etc.) and over 10 cm. Chen et al. [11] used Mask R-CNN [12] to predict an ETT and a tracheal bifurcation for each image at first, and then found the ETT tip and Carina by a feature extraction post-process. Their method achieves a state-of-the-art performance. Although these CNN-based methods eased the feature extraction process, the context and semantic feature representation ability of these methods are still not enough. Therefore, to further lessen the burden on researchers and doctors, we need to improve these exciting methods.

Considering the efficiency and the flexibility of a model, in this paper, an end-to-end architecture was proposed, which was composed of FCOS: Fully Convolutional One-Stage Object Detection [13] and an attention mechanism called Coarse-to-Fine Attention (CTFA). FCOS was a one-stage anchor-free object detection method based on four distance values between the predicted center point and four sides of a bounding box. Furthermore, CTFA was applied to the deepest layer of backbone in FCOS, which consisted of global-modelling attention and scale attention. CTFA captured the long-range relationships between features by global-modelling attention based on [14] and rescaled the feature values with local relationships by scale attention based on [15]. Moreover, a mask prediction branch was attached during training, keeping more useful information. After finding the candidates of ETT tips and Carinas, a post-process algorithm was utilized to decide the best feature point of the ETT tip and Carina. The experiments on the chest radiographs datasets provided by ICU in National Cheng Kung University Hospital and Tainan Hospital showed that our method outperformed the existing state-of-the-art models. In summary, the main contributions of this paper are summarized as follows:

1. A new spatial attention (SA) module was proposed, which noticed the feature distribution in spatial and channel domains simultaneously. Different from previous studies, the SA employed the weight of channel attention to refine the weight of spatial attention, which also solved the problem from [15] where it overlooked the synergies between the channel and spatial attention. Moreover, the performance of SA was better than other attention modules.
2. A new merge method of attention modules was proposed, which fused global modelling attention and scale attention. Instead of stacking the same type of attention together, this method enlarged the ability of feature extraction in a coarse-to-fine way and received better results.
3. The architecture proposed by this paper improved the outcomes of object detection tasks by adding a segmentation branch. With the segmentation branch, the architecture kept more useful information during the feature extraction.
4. The experimental result demonstrated that the proposed architecture in this paper achieves outstanding performance on the chest radiograph datasets provided by the ICU in National Cheng Kung University Hospital and Tainan Hospital.

The rest of this paper is organized as follows. Section 2 introduces previous research about object detection and attention module. Section 3 explains the proposed method for detecting endotracheal intubation malposition. Section 4 shows and discusses the experimental results. Section 5 summarizes this paper.

## 2. Related Works

### 2.1. Object Detection

Object detection is a subfield of computer vision. The purpose of object detection is to localize and recognize objects with bounding boxes; this is different from classification, which is aimed at classifying a view into a class. Generally, it can be separated into one-stage detectors and two-stage detectors, based on whether the region proposal is adopted or not. Moreover, object detection can also be separated into anchor-based detectors and anchor-free detectors based on whether predefined anchors are employed or not. This paper used a one-stage anchor-free architecture named FCOS [13] as the base.

#### 2.1.1. Anchor-Based Approach

Anchor-based detectors set anchor boxes in each position on the feature map and predict the probability of having objects in each anchor box. This approach can be separated into two classes by whether it uses region proposals or not, i.e., two-stage detector and one-stage detector. Two-stage detectors generate region proposals and then classify and refine them. For instance, Ren et al. [16] improved Fast R-CNN [17] by proposing a region proposal network (RPN), which generated high-quality region proposals with more efficiency than the Selective Search applied in Fast R-CNN. After finding the proposals, they used the region of interest (ROI) pooling to produce feature maps with the same resolution for further refinement. Cai et al. [18] noticed the impact of intersection over union (IoU) threshold when training object detector. Therefore, they proposed Cascade R-CNN, which consists of a sequence of detectors trained with an increasing IoU threshold. He et al. proposed Mask R-CNN [12] which is based on Faster R-CNN [16] and Feature Pyramid Network (FPN) [19], trying to perform object detection and segmentation at the same time.

One-stage detectors classify and regress the location directly. These detectors are more efficient and lightweight but have lower performance compared to two-stage detectors. For instance, SSD [20] detects objects of different scales by different layers in VGG-16 [21], i.e., small objects are detected on high-resolution feature maps, and large objects are detected on low-resolution feature maps. Liu et al. [22] proposed RFBNet to strengthen the receptive fields (RFs) based on SSD and the structure of the RFs in the human visual system. The RF Block (RFB) in RFBNet is similar to a mix of the inception block [10,23] and the atrous spatial pyramid pooling (ASPP) [24]. Lin et al. proposed Focal Loss to solve the extreme imbalance between positive and negative samples during training [25]. The Focal Loss which is similar to weighted cross entropy (CE) loss provided larger weights for positive samples and lower weights for negative samples. Based on this loss function, Lin et al. also proposed a one-stage detector named RetinaNet which used ResNet [26] and FPN [19] as a backbone to demonstrate the capability of Focal Loss.

#### 2.1.2. Anchor-Free Approach

Anchor-free detectors do not need to design anchor boxes, avoiding the complicated computations related to anchor boxes. FCOS [13,27] transformed object detection tasks into per-pixel prediction tasks and used multi-level prediction to improve the recall. Based on their approach, they regressed the distance between the location and the four sides of the bounding box. Furthermore, they also proposed the center-ness branch to suppress the low-quality detected bounding boxes. FoveaBox [28] is similar to FCOS. FoveaBox also applied FPN to solve the intractable ambiguity which is caused by the overlaps in ground truth boxes. Additionally, they also regressed the offset between the location and the four sides of the bounding box. However, the definition of the positive samples in FoveaBox is different from FCOS. They used a formula with a shrunk factor to form shrunk ground truth boxes. If a sample falls into the shrunk ground truth boxes, the sample is positive, otherwise, it is negative. Moreover, the formula for offset computation is also different from FCOS.

CornerNet [29] detected an object as a pair of the top-left corner and bottom-right corner of a bounding box with heatmaps and then grouped the pairs of corners belonging to the same instance with embedding vectors. Furthermore, to solve the problem that the corner outside the object cannot be localized based on local evidence, they proposed corner pooling which took in two feature maps to encode the locations of the corner. During training, they used unnormalized 2D Gaussian to ease the difficulty of detecting the exact position of a corner. Duan et al. noticed that the CornerNet had a high false-positive rate. They intuited that the higher IoU of bounding boxes, the higher probability of the center key points in the central region will be predicted as the same class [30]. Therefore, they improved CornerNet with two strategies named center pooling and cascade corner pooling and represented each object using a center point and a pair of corners. They defined a scale-aware central region for each bounding box, and the bounding box will be preserved if the center key point had the same class as the bounding box that was detected in its central region.

### 2.2. Attention Mechanism

Attention mechanisms that come from Natural Language Processing (NLP) make a huge impact on vision tasks. Generally, attention mechanisms are adopted to determine where to focus. They take feature maps as input and produce weights for each value on the feature maps. The weights could be formed by the relationships between two pixels, the relationships between channels, spatial relationships, or unmentioned relationships. Attention mechanisms can be separated into dot-product attention, channel attention, spatial attention, level attention, and some variants by the relationships they adopt. Moreover, attention mechanisms can also be separated into global modelling attention and scale attention by their purpose [31,32]. Therefore, this paper classifies the attention mechanisms from this point of view.

#### 2.2.1. Global Modelling Attention

Global modelling attention is effective for modelling the long-range global contextual information. Wang et al. proposed a non-local module to capture long-range dependencies with deep neural networks in computer vision [33]. Fu et al. proposed a Dual Attention Network (DANet) to capture feature dependencies in the spatial and channel dimensions with two parallel attention modules [34]. One is a position attention module and the other is a channel attention module. Both of these modules are similar to the non-local module. Miao et al. proposed CCAGNet [14] that considered multi-region context information simultaneously by combining Cross-context Attention Mechanism (CCAM), Receptive Field Attention Mechanism (RFAM), and Semantic Fusion Attention Mechanism (SFAM). The CCAM modeled the long-range and adjacent relationships among the feature values by two parallel non-linear functions. Moreover, the RFAM was proposed to improve the RFB in RFBNet [22] by inserting channel attention and spatial attention implemented with CCAM. Finally, SFAM was proposed to improve traditional up-sampling by Sub-Pixel convolution up-sampling [35] followed by CCAM.

#### 2.2.2. Scale Attention

Scale attention is adopted to determine where to focus and suppress uninformative features. This attention mechanism is widely used in computer vision. Hu et al. proposed Squeeze-and-Excitation Networks (SENet) [36] which consist of a squeezing process and an excitation process. The squeezing process used global average pooling to generate the relationships between channels. Then, in the excitation process, the relationships mentioned above pass through a bottleneck with two fully connected layers to fully capture channel-wise dependencies. Woo et al. proposed CBAM to capture channel-wise relationships and spatial-wise relationships in order [15]. Their channel attention module included two parallel SE blocks with max pooling and average pooling. Their spatial attention module also used max pooling and average pooling to grab the relationships in spatial domains. Gu et al. aggregated spatial attention, channel attention, and level attention together and proposed Comprehensive Attention Neural Networks (CA-Net) [37]. Their spatial attention included a non-local block and the two-pathway attention blocks which refined the deep feature maps with the shallow ones. Moreover, their level attention consisted of channel attention and refinement process, which integrated each scale output in the decoder.

## 3. Materials and Methods

### 3.1. Overview

**Figure 1:**
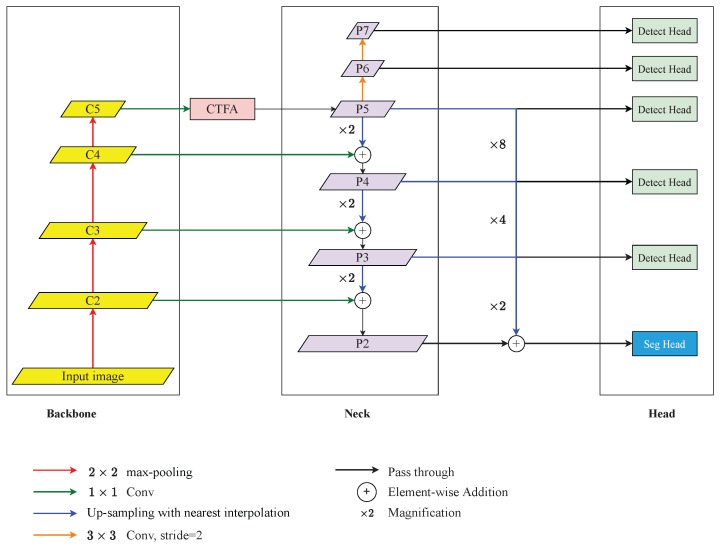
An overview of the proposed architecture for detecting the malposition. It consists of a ResNet-based backbone, Coarse-to-Fine Attention (CTFA), FPN-based neck, FCOS-based detection head, and segmentation head. The legends below demonstrate the operations above.

The proposed overall architecture is shown in Figure 1. In general, the proposed method embedded CTFA and a mask branch into FCOS [13] and employed the ResNet50 [26] as a backbone. This architecture aimed to take chest radiographs as input and located the ETT tip and the Carina. After finding the candidates of ETT tips and Carinas, a post-process algorithm was applied to refine the best ETT tip and Carina. ResNet50 [26] without the last down-sampling operations was applied to extract semantic features and the feature maps from high resolution to low resolution are denoted as $\left\{C_{2-5}\right\}$, respectively. Followed by $C_{5}$, CTFA was applied to refine the feature representation and then passed into the neck. The neck was implemented by FPN [19] which up-sampled the low-resolution feature maps and merged them with the high-resolution feature maps by element-wise summation. The feature maps produced by FPN are defined as $\left\{P_{2-5}\right\}$ from high resolution to low resolution. Additionally, the $P_{5}$ would pass through a series of convolutions with strides of 2 and kernel sizes of 3×3 to form $P_{6}$ and $P_{7}$. Afterwards, $\left\{P_{3-7}\right\}$ were fed into the FCOS detection head, and $\left\{P_{3-5}\right\}$ were also fed into a segmentation head with $\left\{P_{2}\right\}$. After detecting where the ETT tip and Carina might be, the post-process algorithm would choose the best ETT tip and Carina feature point through Gaussian masks generated from the center of the predicted bounding boxes of ETT and tracheal bifurcation.

### 3.2. Coarse-to-Fine Attention (CTFA)

**Figure 2:**
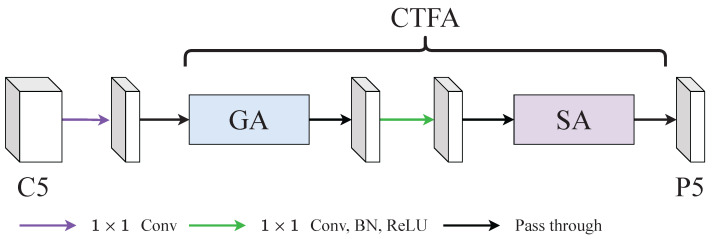
An illustration of coarse-to-fine attention (CTFA). CTFA consisted of a global-modelling attention (GA) and a scale attention (SA). GA was aimed at capturing long-range relationships and SA was aimed at reweighting with local relationships.

CTFA fused global-modelling attention (GA) and scale attention (SA) with a point-wise convolution as shown in Figure 2. Concretely, the feature map produced by GA would pass through a point-wise convolution to smooth the features. Then, these smoothed feature maps were fed into SA to rescale features. We found that the Cross-context Attention Mechanism (CCAM) in [14] can grab long-range relationships and its performance was good as shown in the Section 4.5.3 of the ablation study. Therefore, we adopted CCAM as our GA in this paper. Moreover, we noticed that the CBAM can grab local relationships. However, some shortages limit the performance of CBAM. Therefore, we addressed these shortages and propose a new SA based on the CBAM.

#### 3.2.1. Global-Modelling Attention (GA)

**Figure 3:**
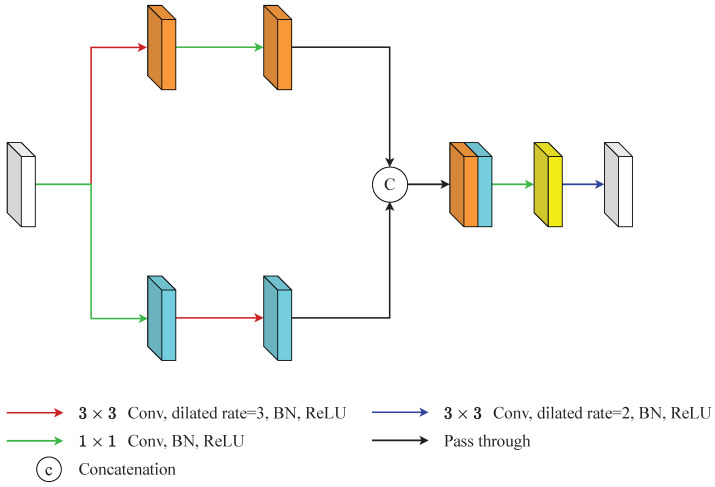
An illustration of global-modelling attention (GA). GA generated long-range relationships through two branches. The upper branch was aimed at capturing long-range context information and the lower branch was aimed at grabbing local context information. Then, this information is integrated by a series of operations.

The CCAM in [14] encoded the cross-region relationship and adjacent-region relationships by two branch. Then, it fused the relationships by concatenation and adopted a series of convolutions followed by a softmax activation function to grab the attention weight. However, we noticed that the performance of CCAM without employing a softmax activation function was better than the performance of CCAM with a softmax activation function in this paper as shown in the Section 4.5.1 of the ablation study. Therefore, GA did not adopt the softmax activation function and the following steps in the CCAM. The architecture of GA is shown in Figure 3 and it can be summarized as:

(1) $$\begin{array}{cc} \; & F_{cr}=Conv_{1\times 1}\left(DiConv_{3\times 3,3}\left(F_{in}\right)\right),\\ \; & F_{ar}=DiConv_{3\times 3,3}\left(Conv_{1\times 1}\left(F_{in}\right)\right), \end{array}$$

(2) $$F_{GA}=DiConv_{3\times 3,2}\left(Conv_{1\times 1}\left(F_{cr}\|F_{ar}\right)\right),$$

where the ReLu and batch normalization are neglected, $F_{in}\in R^{C\times H\times W}$ denotes the input feature map, DiConv denotes the dilated convolution and the parameters are the kernel size and the dilation rate, respectively, Conv denotes the traditional convolution and the parameter is the kernel size, $F_{cr}$ and $F_{ar}$ denote the feature maps generated from the cross-region branch and the adjacent-region branch, ‖ denotes the concatenate operation, $F_{GA}$ represents the output of the GA module.

Specifically, GA generated long-range relationships through two branches. The upper branch $F_{cr}$ captured long-range context information by a dilated convolution whose kernel size was 3×3 and the dilation rate was 3, and smooths the features by a point-wise convolution (Equation (1)). The lower branch $F_{ar}$ grabbed local context information by a point-wise convolution and expands the influence of the features by a dilated convolution whose kernel size was 3×3 and the dilation rate was 3 (Equation (1)). These two feature maps concatenated to form the feature maps $F_{ga}=F_{cr}\|F_{ar}\in R^{2C\times H\times W}$. Then, $F_{ga}$ passed through a point-wise convolution and a dilated convolution whose kernel size was 3×3 and the dilation rate was 2 to produce the final output of GA, $F_{GA}$ (Equation (2)). The point-wise convolution here was used to decrease the channel numbers and the dilated convolution was used to expand the influence of features again.

#### 3.2.2. Scale Attention (SA)

**Figure 4:**
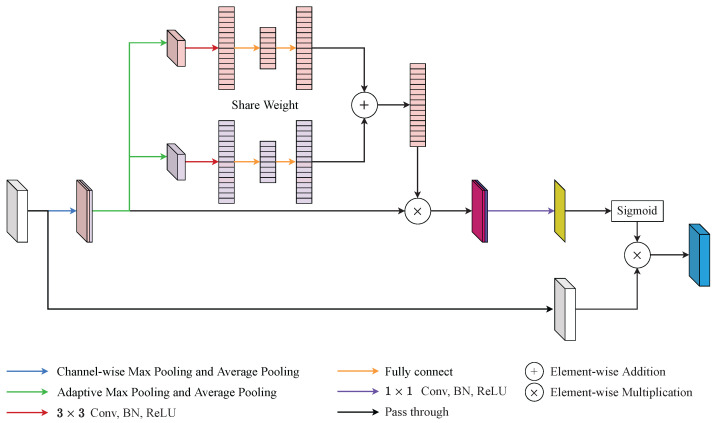
An illustration of scale attention (SA). SA addressed the defects of convolutional block attention module (CBAM) by adaptive channel pooling and squeeze-and-excitation (SE) block.

There were certain problems with the use of CBAM [15]. One of these was that CBAM ignored the interaction between channel attention and spatial attention. Another was that the channel-wise pooling operations overlooked the feature distribution of the feature maps. Based on the above analysis, the SA was proposed to smooth out these defects at the same time. The construction of SA is shown in Figure 4 and its overall process can be summarized as:

(3) $$F_{s}=M_{cavg}\left(F_{x}\right)\|M_{cmax}\left(F_{x}\right),$$

(4) $$\begin{array}{c} F_{SE1}=fc_{ex}\left(fc_{sq}\left(Conv_{3\times 3}\left(M_{aavg}\left(F_{s}\right)\right)\right)\right),\\ F_{SE2}=fc_{ex}\left(fc_{sq}\left(Conv_{3\times 3}\left(M_{amax}\left(F_{s}\right)\right)\right)\right), \end{array}$$

(5) $$F_{sa}=Conv_{1\times 1}\left(F_{s}⊗\left(F_{SE1}⊕F_{SE2}\right)\right),$$

(6) $$F_{SA}=F_{x}⊗\left(\sigma \left(F_{sa}\right)\right),$$

where the ReLu and batch normalization are neglected, $F_{x}$ denotes the input feature map, $M_{cavg}$ and $M_{cmax}$ denote channel-wise average pooling and channel-wise max pooling, ‖ denotes the concatenate operation, $F_{s}$ denotes the feature map after channel-wise pooling, $M_{aavg}$ and $M_{amax}$ denote adaptive average pooling and adaptive max pooling, Conv denotes the convolutional layer and the subscript represents the kernel size, $fc_{sq}$ and $fc_{ex}$ are two fully connected layers, $F_{SE1}$ and $F_{SE2}$ denote the feature maps in two branches after two fully connected layers, ⊕ denotes the element-wise summation, $F_{sa}$ represents the spatial weight, σ represents the softmax function, $F_{SA}$ represents the output of the SA module.

Specifically, SA can be separated into four steps. First, the input feature map $F_{x}\in R^{C\times H\times W}$ was fed into two channel-wise poolings and generated two feature maps $F_{cavg}=M_{cavg}\left(F_{x}\right)\in R^{8\times H\times W}$ and $F_{cmax}=M_{cmax}\left(F_{x}\right)\in R^{8\times H\times W}$, respectively. Afterwards, $F_{cavg}$ was concatenated with $F_{cmax}$ to generate $F_{s}\in R^{16\times H\times W}$ as the Equation (3). By preserving more pooled channels, SA kept more of the channel information of the feature maps. Second, $F_{s}$ passed into adaptive max pooling and adaptive average pooling simultaneously to create $F_{amax}=M_{amax}\left(F_{s}\right)\in R^{16\times 3\times 3}$ and $F_{aavg}=M_{aavg}\left(F_{s}\right)\in R^{16\times 3\times 3}$, respectively. Then, $F_{amax}$ and $F_{aavg}$ would pass through one shared-weight convolution layer and two shared-weight fully connected layers to generate $F_{SE1}$ and $F_{SE2}$ as the Equation (4). By retaining a higher resolution, SA saved more spatial information than the traditional pooling method. Third, SA directly fused $F_{SE1}$ and $F_{SE2}$ with a element-wise summation. Thereafter, the mixed feature was element-wise multiplied with $F_{s}$ followed by a point-wise convolution layer as Equation (5). This step integrated channel attention weight into the spatial attention process and reduced the channel of the merged features with a point-wise convolution layer. Finally, the spatial attention feature, $F_{sa}\in R^{1\times H\times W}$, passed through softmax to grab the spatial attention weight. Then, the output of SA, $F_{SA}\in R^{C\times H\times W}$, was generated from the element-wise multiplication with $F_{x}$ and the spatial attention weight as the Equation (6).

### 3.3. Mask Branch

We noticed that the Mask R-CNN with a special feature extraction method proposed by Chen et al. [11] refined the feature points of the ETT tip and the Carina with the mask of ETT and tracheal bifurcation and achieved state-of-the-art performance. Inspired by their feature extraction algorithm and considering the characteristics of FCOS [13], this paper adopted a mask branch into the neck, trying to refine the feature point of the ETT tip and Carina in the same way. The architecture of the mask branch is shown in Figure 1 and its operation can be summarized as:

(7) $$F_{seg}=P_{2}⊕Upsameple_{2}\left(P_{3}\right)⊕Upsameple_{4}\left(P_{4}\right)⊕Upsameple_{8}\left(P_{5}\right),$$

(8) $$F_{SEG}=Conv_{3\times 3}\left(Upsample_{4}\left(Conv_{3\times 3}^{4}\left(F_{seg}\right)\right)\right),$$

where the ReLu and batch normalization are neglected, $Upsample_{i}$ denotes enlarging the resolution *i* times with nearest interpolation, ⊕ denotes the element-wise summation, $F_{seg}$ denotes the feature map after fusion, $Conv_{j}^{i}$ denotes stacking convolution with *j* kernel size *i* times, $F_{SEG}$ denotes the final output of the segmentation head.

Considering that the features of an object might be separated into the feature pyramid, and a large magnification may cause some problems, this paper only fused $P_{3-5}$ together (Equation (7)). Explicitly, $P_{3-5}$ would be enlarged to the same resolution with $P_{2}$ and then summarized together. Afterwards, the fused feature maps would feed into the segmentation head which includes a series of convolution and up-sampling to generate the segmentation mask (Equation (8)).

### 3.4. Post-Process Algorithm

**Figure 5:**
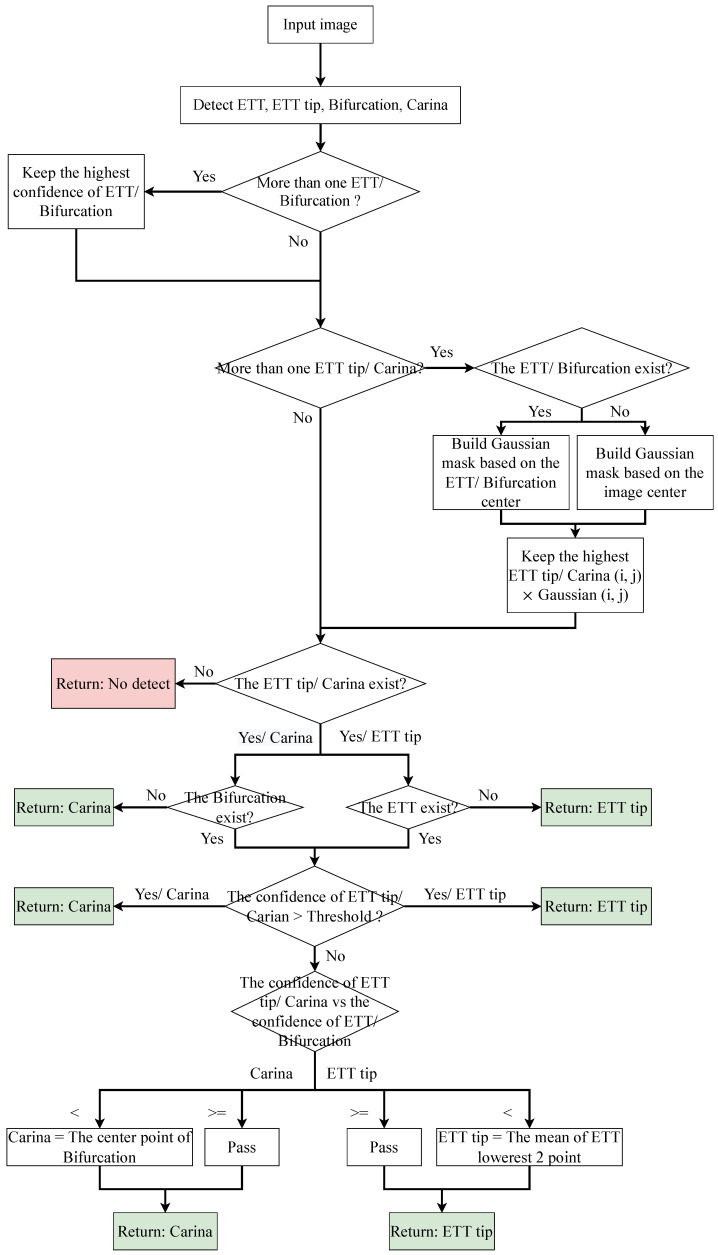
An illustration of post-process.

The process of the post-process algorithm is shown in Figure 5. In general, the algorithm has three steps. First, it found the highest confidence bounding box (bbox) of the ETT/tracheal bifurcation. Second, it applied a Gaussian mask based on the center of the ETT/tracheal bifurcation bbox or the center of the image to determine the best ETT tip/Carina. Finally, if the confidence of the best ETT tip/Carina was less than a threshold, then the best ETT tip/Carina would be generated after comparing with the confidence of the ETT/tracheal bifurcation bbox.

Expressly, in the first step, the algorithm kept the first bbox of the ETT/tracheal bifurcation as the best one and enumerated all bboxes which were classified as ETT/tracheal bifurcation. If the confidence of a bbox was larger than the best one, then the best bbox would be replaced by the bbox. As for the second step, if the bbox of ETT/tracheal bifurcation existed, the algorithm would produce a Gaussian mask based on the center of the bbox. Otherwise, the Gaussian mask would be generated based on the center of an input image. Afterwards, the confidence of the bboxes classified as ETT tip/Carina would multiply the value which was grabbed from the same position as the center point of the bbox on the Gaussian mask. Then, the algorithm chose the best ETT tip bbox/Carina bbox as the first step based on this multiplied value. After checking whether the ETT tip bbox/Carina bbox existed or not, the algorithm adopted a threshold to filter out the ETT tip bboxes/Carina bboxes which did not need to be purified. Finally, the remaining bbox of ETT tip/Carina was compared with the corresponding bbox of ETT/tracheal bifurcation. If the confidence of the ETT tip bbox was lower than the ETT bbox, the ETT tip would be replaced by the center point of the lowest two points of the ETT bbox. Otherwise, the ETT tip was the center point of the ETT tip bbox. As for Carina, if the confidence of the Carina bbox was lower than the tracheal bifurcation bbox, the Carina would be replaced by the center point of the tracheal bifurcation bbox. Otherwise, the Carina was the center point of the Carina bbox.

### 3.5. Loss Function

The loss function was based on FCOS [13] appended with a segmentation loss. The formula of the loss function can be summarized as:

(9) $$L_{total}=L_{cls}+L_{reg}+L_{centerness}+L_{seg},\;$$

where the $L_{cls}$ is the classification loss implemented with focal loss [25], $L_{reg}$ is the regression loss implemented with GIoU loss [38], $L_{centerness}$ is the center-ness loss implemented with binary cross entropy loss [13], $L_{seg}$ is the segmentation loss implemented with cross entropy loss plus dice loss as in [39].

(10) $$\begin{array}{cc} \; & L_{cls}=FL\left(p_{t}\right)=-a_{t}{\left(1-p_{t}\right)}^{\gamma }log\left(p_{t}\right),\\ \; & p_{t}=\left\{\begin{array}{cc} p, & if\;y=1\\ 1-p, & otherwise, \end{array}\right.\\ \; & a_{t}=\left\{\begin{array}{cc} a, & if\;y=1\\ 1-a, & otherwise, \end{array}\right.\; \end{array}$$

The formula of focal loss is shown in Equation (10), where y∈{±1} denotes the ground-truth class, p∈[0,1] denotes the predicted probability for the class with label y=1, a∈[0,1] denotes a weighting factor for class with label y=1. Additionally, the γ is set to 2 as [13] did. This loss function can mitigate the class imbalance problem by down-weight easy samples.

(11) $$\begin{array}{cc} \; & L_{reg}=L_{GIoU}=1-GIoU\\ \; & GIoU=IoU-\frac{\left|\begin{array}{c} C\;\backslash \;(A\cup B) \end{array}\right|}{\left|\begin{array}{c} C \end{array}\right|}\\ \; & IoU=\frac{\left|\begin{array}{c} A\cap B \end{array}\right|}{\left|\begin{array}{c} A\cup B \end{array}\right|} \end{array}$$

The formula of *GIoU* loss is shown in Equation (11), where A,B denotes two arbitrary shapes, *C* denotes the smallest convex shapes enclosing both *A* and *B*. *GIoU* solved the problem that if $\left|A\cap B\right|=0$, *IoU* cannot reflect the relationship between *A* and *B*.

(12) $$\begin{array}{cc} \; & L_{centerness}=L_{BCE}\left(centerness,centerness^{*}\right)\\ \; & centerness^{*}=\sqrt{\frac{min(l^{*},r^{*})}{max(l^{*},r^{*})}\times \frac{min(t^{*},b^{*})}{max(t^{*},b^{*})}} \end{array}$$

The $L_{BCE}$ is the binary cross-entropy loss, the $centernss^{*}\in \left[0,1\right]$ denotes the ground-truth value of centerness, $l^{*},r^{*},b^{*},t^{*}$ denotes the distance from a feature point to the left, right, top, and bottom of the relative ground-truth bbox. FCOS down-weighted the confidence of low-quality predicted bboxes by adding this loss function. Concretely, during the inference phase, the classification score will multiply with the corresponding predicted centerness score. Thus, the low-quality bboxes could be filtered out by non-maximum suppression (NMS).

(13) $$\begin{array}{cc} \; & L_{seg}=L_{Dice}+\lambda L_{CE}\\ \; & L_{Dice}=1-Dice\\ \; & Dice=\frac{2\left|A\cap B\right|}{\left|A\right|+\left|B\right|}\\ \; & L_{CE}\left(p_{t}\right)=-log\left(p_{t}\right)\\ \; & p_{t}=\left\{\begin{array}{cc} p, & if\;y=1\\ 1-p, & otherwise, \end{array}\right. \end{array}$$

The λ is a trade-off between Dice loss and cross-entropy loss, and is set to 1 in all our experiments, the $L_{Dice}$ is the dice loss, A,B denotes two arbitrary shapes as in Equation (11), $L_{CE}$ denotes the cross-entropy loss, and p∈[0,1] denotes the predicted probability for the class with label y=1 as the Equation (10).

## 4. Results and Discussion

### 4.1. Dataset and Evaluation Metrics

**Figure 6:**
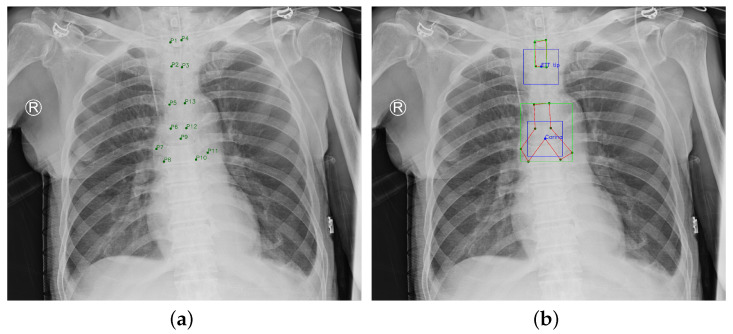
Ground Truth. (**a**) Original ground truth. (**b**) Pre-processed ground truth.

This paper was approved by the institutional review board (IRB) of the National Cheng Kung University (NCKU) Hospital (IRB number: A-ER-108-305). The chest radiograph dataset provided by NCKU Hospital includes 1,870 portable chest radiographs of intubated ICU patients in DICOM format and the ground truth (GT) annotations were labeled by two board-certified intensivists. The GT ETT was labelled by four points ($P_{1-4}$), and the GT tracheal bifurcation was labelled by nine points ($P_{5-13}$), as shown in Figure 6a. The purpose of this paper was to detect the malposition by locating the ETT tip and the Carina. Therefore, this paper adopted two boxes with a size of 300×300 to label the feature point of the ETT tip which was the middle point of $P_{2}$ and $P_{3}$, and the feature point of the Carina which was $P_{9}$ (the feature point of ETT tip and Carina are at the center of the boxes). Furthermore, the 13 points were corrected to be sequential for generating the GT mask. In summarize, the GT became Figure 6b. In Figure 6b, the green nodes denote the original points labeled by the intensivists, the blue nodes denote the ETT tip and Carina, the green boxes denote the GT bboxes of ETT and tracheal bifurcation, the blue boxes denote the GT bboxes of ETT tip and Carina, and the red polygons denote the GT mask of ETT and tracheal bifurcation. Finally, this paper used extra 150 chest radiographs to validate the proposed approach.

This paper applied object error, distance error, recall, precision, and accuracy to evaluate the performance. Object error and distance error were implemented with the Euclidean Distance. The object error of the ETT tip was defined as the distance between the center of the predicted ETT tip bbox and the center of the GT ETT tip bbox. The object error of the Carina was defined as the distance between the center of the predicted Carina bbox and the center of the GT Carina bbox. Moreover, the distance between the ETT tip and the Carina was named the ETT–Carina distance. The distance error was defined as the absolute difference between the GT ETT–Carina distance and the predicted ETT–Carina distance. As for recall and precision, this paper defined successful detection (true positive) as when the object error was no more than 10 mm [40], a false positive indicated that a region was a specific object but the region did not include the object, and a false negative was that the model did not indicate the GT object. Therefore, the recall and precision are defined as:

(14) $$\mathrm{Recall}=\frac{TP}{\left(TP+FN\right)},$$

(15) $$\mathrm{Precision}=\frac{TP}{\left(TP+FP\right)},$$

(16) $$\mathrm{Accuracy}=\frac{\left(TP+TN\right)}{\left(TP+TN+FP+FN\right)}$$

Moreover, by the Goodman’s criteria [41,42], the ideal distance of the ETT–Carina was in the range of [30, 70] mm. However, another paper [43] recommended that the average correct tube insertion depth was 21 cm in women and 23 cm in men. In this situation, the distance of the ETT–Carina was less than 25 mm. Taking this into consideration, this paper defined that the endotracheal intubation was suitable if the distance of the ETT–Carina is in the range of [20, 70] mm, as in [9].

### 4.2. Implementation Details

The architecture was implemented based on Pytorch. During training, Adam [44] with the initial learning rate of $10^{-4}$ was adopted. The batch size was set to 2 and the total epochs were set to 120. Moreover, we employed a warm-up policy for the first five epochs and utilized the cosine learning rate decay policy for the rest of the epochs. Additionally, the input images were resized to have their shorter side be 800 and their longer side less or equal to 1333. In addition, random color jitter, random rotation angle within the range of [−10,10] degrees, and random cropping were used during training. Furthermore, the dataset provided by NCKU Hospital was equally divided into five folders to execute 5-fold cross-validation. Finally, all of the experiments were executed on an Nvidia GeForce RTX 2080 Ti GPU.

### 4.3. Results

As shown in Table 1, by the definition of “suitable position”, this paper achieved 88.82% accuracy on the NCKU dataset and 90.67% on the external validation based on the annotations from board-certified intensivists. Moreover, the mean of the ETT–Carina distance error was 5.333 mm and the standard deviation of the ETT–Carina distance error was 6.240 mm. In [9], the mean of the ETT–Carina distance error was 6.9 mm, and the standard deviation of the ETT–Carina distance error was 7.0 mm. Table 2 shows that the distance error of 85.83% images were less than 10 mm on the NCKU dataset and 84.00% images were less than 10 mm on the external validation. Table 3 and Table 4 show the confusion matrix of diagnosis in NCKU dataset and external validation.

For the ETT tip and the Carina, Table 5 shows that the recall and precision of the ETT tip were 90.96% and 91.60%, separately. The recall and precision of the Carina were 93.90% and 94.10%, separately. Table 6 shows the mean and standard deviation of object error on the ETT tip were 4.304 mm and 5.526 mm, respectively. The mean and standard deviation of object error on the Carina were 4.118 mm and 3.655 mm, respectively. Table 7 shows that in the object error of the ETT tip, there were 90.96% images not longer than 10 mm. Table 8 shows that in the object error of the Carina, the differences of the 93.90% images were not longer than 10 mm. Although most of the performances on the external validation were dropped, the performance on ETT–Carina still showed the robustness of our architecture in detecting the malposition of the ETT.

### 4.4. Compare with the SOTA

This section compares the proposed method with the state-of-the-art (SOTA) method proposed by Chen et al. [11]. Both of these methods executed the experiment on the same datasets. Table 9 presents the accuracy of detecting malposition and the distance error of the ETT–Carina, and Table 10 presents the error distribution of the ETT-Carina distance. Furthermore, the percentage in the brackets denotes the improvement or degeneration rate of the performances. Based on the tables, we observed that most of the performances of the proposed method were better than SOTA. It is worth mentioning that the proposed method had higher accuracy on both data sets, which indicated that the proposed method could be more accurate in detecting the malposition of the ETT.

Table 11 and Table 12 demonstrate the performance of the object error and the error distribution on the ETT tip. Table 13 and Table 14 demonstrate the performance of the object error and the error distribution on the Carina. In general, our method was bad at detecting the ETT tip but good at detecting the Carina compared with SOTA. However, the improvement on the Carina could cover the degeneration of the ETT tip. Therefore, our method could detect the malposition more effectively.

### 4.5. Ablation Study

The ablation studies were performed on the NCKU dataset with folder 5 as the testing data to verify the effectiveness of our method. This section only focuses on the accuracy, the mean distance error, and the standard deviation.

#### 4.5.1. Structure of GA

Table 15 demonstrates the performance of whether the GA adopted a softmax function. This results presented that the GA without a softmax activation function achieved a better outcome. Therefore, CTFA employed GA without a softmax function.

#### 4.5.2. Structure of SA

In Table 16, the *c* and *k* in the brackets behind the SA denote the pooled channel number and the followed kernel size. The SE in the brackets behind the SA shows whether the SA adopts a SE block as the channel attention. From the six rows above, we observed that when the output channel number of the channel-wise max pooling and average pooling was 8, and the followed kernel size was 1, the performance was the best. Therefore, CTFA employed SA with 8 pooled channel numbers followed by a point-wise convolution. Furthermore, comparing the fourth row with the last row, we noticed that employing the SE block in SA would increase the performance.

#### 4.5.3. Compare with Attention Modules

Table 17 and Table 18 display the results of adopting existing attention modules in FCOS [13]. This paper calculated the number of parameters and giga floating point operations (GFLOPs) for each attention module with an image size of 224×224. Furthermore, the percentage in the brackets at the “Parameter (M)” and “GFLOPs” column is the degree of increment compared with FCOS. By Table 17, we discovered that the SE block [36], Nonlocal block [33], Nonlocal with CSP block [45] and the CBAM block [15] brought no benefit to the accuracy of ETT-Carina, but the CCAM block [14] and the SA block proposed by this paper could improve the accuracy. Moreover, the SA block was more lightweight than the CCAM block and achieved higher accuracy, as shown in Table 18. These results illustrated the efficiency and effectiveness of the SA block.

#### 4.5.4. Fusion Method

This part explores the fusion method. The second row in Table 19 denotes that the $C_{5}$ with reduced channel directly passes through SA, followed by GA. The third row denotes that the $C_{5}$ with reduced channel goes through SA and GA in a parallel fashion, and then concatenates the output feature maps together followed by a point-wise convolution, batch normalization, and relu to integrate the feature map. The last row denotes that the $C_{5}$ with reduced channel directly passes through GA, followed by SA. As shown in Table 19, the last row demonstrated that the long-range relationship rescaled by the local relationship could improve the performance. Moreover, this was the best connection method in our ablation studies; thus, the CTFA adopted this method to grab better features in this paper.

#### 4.5.5. Fusing Global Modelling Attention and Scale Attention

Table 20 presents the result of fusing a global modelling attention and a scale attention. Here, ${}^{*}2$ denotes stacking two of the same attention module together. We observed that when directly stacking two of the same attention module together, the accuracy would drop compared with the performance in Table 17. Specifically, when stacking two CSPnonlocal blocks together, the accuracy would degrade from 86.10% to 85.56%, and when stacking two CCAM blocks together, the accuracy would degrade from 86.90% to 86.10%. Furthermore, stacking two SA blocks also grabbed a worse result. However, if we stacked global modelling attention with scale attention, the performance would increase, as shown in the last two rows. This result showed that the global modelling attention could grab long-range relationships and the scale attention could refine the attention map effectively. Therefore, stacking these two kinds of attention modules together could achieve higher performance.

#### 4.5.6. Mask Branch

In Table 21, the second row denotes that CTFA is adopted in FCOS, and the “Seg” denotes that the CTFA and the mask branch are adopted in FCOS at the same time. Moreover, the text in the brackets behind “Seg” in the “Method” column denotes which mask annotation our approach employed at the mask branch, and the “Fusion” in the “Method” column denotes that we directly fuse the feature maps from $P_{2}$ to $P_{5}$. Comparing the results of the second row and third row, we noticed that the mask branch did not bring advantages to the detection model. However, if we only used the annotation of ETT and tracheal bifurcation, the performance would be improved. This phenomenon might be caused by the occlusion of the annotations. Concretely, the annotations of the ETT and the tracheal bifurcation were always occluded by the annotation of the ETT tip and the Carina. Therefore, the mask branch might focus on the ETT tip and the Carina which was similar to the original detection model (FCOS + CTFA). However, if we only adopted the annotation of the ETT and the tracheal bifurcation, the detection model might pay more attention to the ETT and the tracheal bifurcation. Thus, the detection model might grab some useful context information for detecting the ETT tip and the Carina. Comparing the last two rows, we found that the fusion policy further improves the performance. Considering the accuracy and the object error, the method of the last row achieved the best result on the internal dataset with folder 5 as the testing data. Moreover, it was the final architecture employed by this paper for detecting the malposition of the ETT.

#### 4.5.7. The Post-Process Algorithm

**Figure 7:**
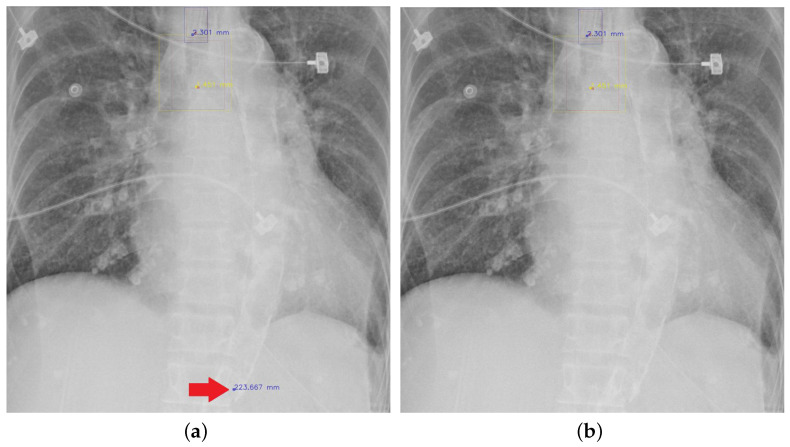
Ensuring at most one ETT tip/Carina left. (**a**) Without post-process. (**b**) With post-process.

**Figure 8:**
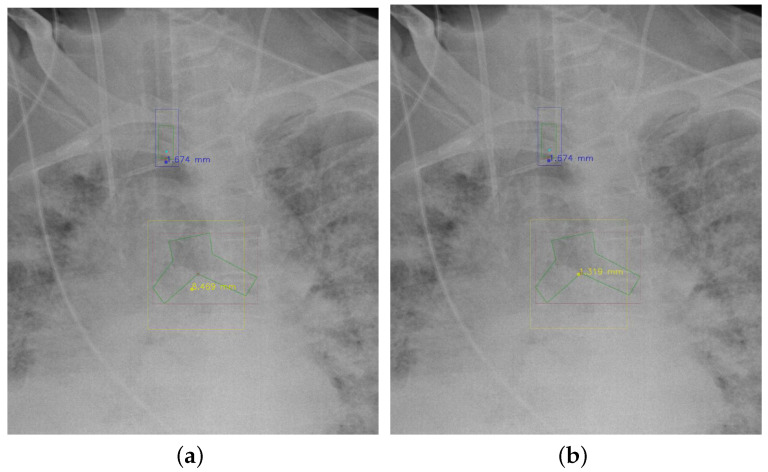
Refining the feature point of ETT tip/Cairna by the bbox of ETT/Bifurcation. (**a**) Without post-process. (**b**) With post-process.

Figure 7 and Figure 8 demonstrate the effect of post-processing. The red bboxes and points in these figures are the GT ETT/bifurcation bboxes and the position of GT ETT tip/Carina, respectively. The green polygon is the GT mask of the ETT and the bifurcation. The blue bbox and point are the predicted ETT bbox and ETT tip, respectively. The yellow bbox and point are the predicted bifurcation and Carina, respectively. Specifically, without the post-process, the model might leave more than one predicted ETT tip/Carina, such as where the red arrow points in Figure 7a. However, with the post-process, the extra points would be removed as shown in Figure 7b. Besides, with the refinement process in the post-process, the feature point of ETT tip/Carina could be further refined as shown in Figure 8. Concretely, the object error of Carina was corrected from 8.469 mm to 1.319 mm.

We observed that the segmentation results of the ETT tip were always near the GT ETT tip. However, although the mask branch could improve the detection result, the segmentation result was not good enough to replace the detection result. As shown in Table 22, if we corrected the detection result of the ETT tip by the segmentation result of the ETT tip when the distance between them was longer than 100 pixels, the performance of ETT would decrease and thus the performance of the ETT-Carina would also decline. Therefore, in the post-process, we only used the bbox result of the ETT and the tracheal bifurcation to refine the position of the ETT tip and the Carina when the ETT tip and the Carina were not good enough.

#### 4.5.8. Visualization

In this part, the red bboxes and points denote the GT ETT/bifurcation bboxes and the position of GT ETT tip/Carina, respectively. The blue bbox and point are the predicted ETT bbox and ETT tip, respectively. The green bbox and point are the predicted bifurcation and Carina, respectively. The light blue point is the position of the ETT tip produced by the mask branch. In the Table 23, the first row demonstrates the good results, the second row shows the medium results, and the third row presents the bad results. We noticed that if an image had a clear location of the ETT tip and the Carina, the performance would be better. Moreover, the life-supporting device might blur the location of the ETT tip and the shadow of the heart might occlude the position of the Carina as shown in the medium case. Apart from the problems mentioned above, the angle of the CXR might also degrade the performance of the proposed method, as shown in the worse results when it is applied.

## 5. Conclusions

This paper proposed an end-to-end architecture to improve the FCOS [13] for detecting the position of the ETT tip and the Carina. First, a Coarse-to-Fine (CTFA) attention module was designed to capture long-range relationships by global-modelling attention (GA) and rescale the feature value with local relationships grabbed by scale attention (SA). Then, a mask branch was adopted to enhance the feature representation of the “backbone” and “neck”. After that, a post-process algorithm was employed to ensure that only one detection result for each class would be left in an image and further refine the feature point of ETT tip and Carina. Experiments on the chest radiograph datasets provided by National Cheng Kung University Hospital demonstrated that the proposed architecture provides an effective solution to improve the performance of the FCOS. The mean malposition accuracy achieved 88.82% and the ETT-Carina distance error was less than 5.333±6.240 mm on the internal dataset. Furthermore, our method indicates the position of the ETT, ETT tip, tracheal bifurcation, and the Carina on an image. Therefore, it is more reasonable for detecting the malposition of the ETT compared with classification-based methods. This end-to-end deep learning-based method could help the intensivists, helping them to keep an eye on the position of the ETT in ICU patients.

Although our proposed methods have exciting results, two shortages still exist that might need to be overcome. First, the annotation of the ETT tip is labelled on the slash at the bottom of the ETT. However, the feature point on the edge is not a good feature point compared with the corner point. Therefore, an accurate ETT tip annotation might further improve the performance. Second, the fusion method adopted for GA and SA is simple, as is the additional mask branch, so further investigations of the interaction between them might improve the performance. In the future, we will focus on these shortages, trying to provide a more reliable model for intensivists. 

## Figures and Tables

**Table 1:** The performance in ETT–Carina distance error.

Test Folder	Acc. (%)	Mean (mm)	Std. (mm)
Folder 1	90.37	5.130	5.609
Folder 2	87.70	5.969	8.325
Folder 3	88.24	5.256	5.491
Folder 4	86.63	5.437	6.663
Folder 5	91.18	4.874	5.111
Average	88.82	5.333	6.240
External val.	90.67	5.015	5.147

**Table 2:** The distance error distribution in ETT–Carina.

Test Folder	≤5 mm (%)	≤10 mm (%)	≤15 mm (%)	≤20 mm
Folder 1	62.57	85.29	93.58	96.79
Folder 2	63.10	84.22	92.25	95.72
Folder 3	63.90	83.96	92.25	95.45
Folder 4	63.90	87.17	92.78	97.06
Folder 5	64.44	88.50	93.85	97.06
Average	63.58	85.83	92.94	96.42
External val.	66.00	84.00	92.67	97.33

**Table 3:** The confusion matrix of diagnosis.

GT	Suitable	Unsuitable
Predict		
Suitable	1350	126
Unsuitable	66	311
Undetection	12	5

**Table 4:** The confusion matrix of diagnosis (external val.).

GT	Suitable	Unsuitable
Predict		
Suitable	110	8
Unsuitable	5	26
Undetection	1	0

**Table 5:** The performance in recall and precision.

Recall and Precision	ETT Tip		Carina	
**Test Folder**	**Recall (%)**	**Precision (%)**	**Recall (%)**	**Precision (%)**
Folder 1	90.64	91.37	94.65	94.91
Folder 2	89.30	89.54	93.58	93.58
Folder 3	90.91	92.14	92.25	92.49
Folder 4	91.18	91.42	94.92	95.17
Folder 5	92.78	93.53	94.12	94.37
Average	90.96	91.60	93.90	94.10
External val.	92.67	93.29	88.00	88.59

**Table 6:** The performance in object error.

Object Error	ETT Tip		Carina	
**Test Folder**	**Mean (mm)**	**Std. (mm)**	**Mean (mm)**	**Std. (mm)**
Folder 1	4.415	5.281	3.952	3.345
Folder 2	4.858	7.869	4.236	3.663
Folder 3	3.974	4.405	4.322	3.947
Folder 4	4.584	6.273	3.895	3.527
Folder 5	3.690	3.800	4.185	3.793
Average	4.304	5.526	4.118	3.655
External val.	3.733	4.613	4.688	4.043

**Table 7:** The object error distribution in ETT tip.

Test Folder	≤5 mm (%)	≤10 mm (%)	≤15 mm (%)	≤20 mm (%)
Folder 1	75.94	90.64	94.39	97.06
Folder 2	75.40	89.30	94.65	96.79
Folder 3	78.61	90.91	95.19	97.06
Folder 4	73.26	91.18	94.92	97.86
Folder 5	81.02	92.78	97.06	97.59
Average	76.85	90.96	95.24	97.27
External val.	83.33	92.67	94.67	96.67

**Table 8:** The object error distribution in Carina.

Test Folder	≤5 mm (%)	≤10 mm (%)	≤15 mm (%)	≤20 mm (%)
Folder 1	74.60	94.65	98.13	99.20
Folder 2	74.06	93.58	97.59	99.20
Folder 3	73.53	92.25	96.52	98.40
Folder 4	78.34	94.92	97.86	98.93
Folder 5	74.06	94.12	98.13	98.40
Average	74.92	93.90	97.65	98.83
External val.	68.67	88.00	96.67	98.00

**Table 9:** The comparison results of accuracy and ETT–Carina distance error.

Method	Malposition Accuracy (%)	ETT-Carina Distance Error	
		Mean (mm)	Std. (mm)
SOTA average [11]	88.11	5.543	6.310
Ours average	88.82 (+0.81%)	5.333 (−3.79%)	6.240 (−1.11%)
SOTA external val. [11]	87.33	5.668	6.651
Ours external val.	90.67 (+3.82%)	5.015 (−11.52%)	5.147 (−22.61%)

**Table 10:** The comparison results of error distribution on the ETT–Carina distance.

Method	ETT-Carina Distance Error Distribution			
	≤5 mm (%)	≤10 mm (%)	≤15 mm (%)	≤20 mm (%)
SOTA average [11]	60.37	84.20	92.78	95.39
Ours average	63.58 (+5.32%)	85.83 (+1.94%)	92.94 (+0.17%)	96.42 (+1.08%)
SOTA external val. [11]	64.00	82.00	90.67	94.67
Ours external val.	66.00 (+3.13%)	84.00 (+2.44%)	92.67 (+2.21%)	97.33 (+2.81%)

**Table 11:** The comparison results of recall, precision, and object error on the ETT tip.

Method	ETT Tip			
	Recall (%)	Precision (%)	Mean (mm)	Std. (mm)
SOTA average [11]	93.31	93.49	4.122	4.402
Ours average	90.96 (−2.52%)	91.60 (−2.02%)	4.304 (+4.42%)	5.526 (+25.53%)
SOTA external val. [11]	90.27	90.27	4.286	5.943
Ours external val.	92.67 (+2.66%)	93.29 (+3.35%)	3.733 (−12.90%)	4.613 (−22.38%)

**Table 12:** The comparison results of error distribution on the ETT tip.

Method	ETT Tip Object Error Distribution			
	≤5 mm (%)	≤10 mm (%)	≤15 mm (%)	≤20 mm (%)
SOTA average [11]	75.08	93.31	96.36	98.21
Ours average	76.85 (+2.36%)	90.96 (−2.52%)	95.24 (−1.16%)	97.29 (−0.94%)
SOTA external val. [11]	79.33	90.27	95.33	96.97
Ours external val.	83.33 (+5.04%)	92.67 (+2.66%)	94.67 (−0.69%)	96.67 (−0.31%)

**Table 13:** The comparison results of recall, precision, and object error on the Carina.

Method	Carina			
	Recall (%)	Precision (%)	Mean (mm)	Std. (mm)
SOTA average [11]	94.70	95.23	4.775	5.342
Ours average	93.90 (−0.84%)	94.10 (−1.19%)	4.118 (−13.76%)	3.655 (−31.58%)
SOTA external val. [11]	91.64	91.96	4.567	4.513
Ours external val.	88.00 (−3.97%)	88.59 (−3.66%)	4.688 (+2.65%)	4.043 (−10.41%)

**Table 14:** The comparison results of error distribution on the Carina.

Method	Carina Object Error Distribution			
	≤5 mm (%)	≤10 mm (%)	≤15 mm (%)	≤20 mm (%)
SOTA average [11]	68.84	94.70	95.55	97.12
Ours average	74.92 (+8.83%)	93.90 (−0.84%)	97.65 (+2.20%)	98.83 (+1.76%)
SOTA external val. [11]	73.33	91.64	95.33	96.54
Ours external val.	68.67 (−6.35%)	88.00 (−3.97%)	96.67 (+1.41%)	98.00 (+1.51%)

**Table 15:** The effect of softmax in GA.

Method	Malposition Accuracy (%	ETT-Carina		ETT Tip		Carina	
		Mean Err. (mm)	Err Std. (mm)	Mean Err. (mm)	Err Std. (mm)	Mean Err. (mm)	Err Std. (mm)
w/softmax	90.11	5.209	6.628	3.968	5.800	4.203	4.097
w/o softmax	91.18	4.911	5.114	3.689	3.802	4.238	3.862

**Table 16:** The effect of channel, kernel and SE block of SA.

Method	Malposition Accuracy (%)	ETT-Carina		ETT Tip		Carina	
		Mean Err. (mm)	Err Std. (mm)	Mean Err. (mm)	Err Std. (mm)	Mean Err. (mm)	Err Std. (mm)
SA (c1 + k7)	83.69	4.904	4.813	3.998	3.625	4.386	3.750
SA (c1 + k1)	85.83	5.648	7.628	4.911	8.605	4.185	3.674
SA (c4 + k1)	85.83	5.182	6.245	4.188	4.067	4.611	5.759
SA (c8 + k1)	87.70	5.067	5.248	4.273	4.418	4.305	4.016
SA (c8 + k7)	83.69	4.644	4.401	4.007	3.615	4.028	3.372
SA (c16 + k1)	85.56	4.883	4.778	3.985	3.696	4.351	3.969
SA (w/o SE)	86.36	5.491	9.697	4.619	11.997	4.391	3.956

**Table 17:** The comparison results of attention modules.

Method	Malposition Accuracy (%)	ETT-Carina		ETT Tip		Carina	
		Mean Err. (mm)	Err Std. (mm)	Mean Err. (mm)	Err Std. (mm)	Mean Err. (mm)	Err Std. (mm)
FCOS [13]	86.10	5.335	7.831	4.254	5.427	4.659	7.497
FCOS + SE [36]	85.03	5.424	5.854	4.284	4.156	4.543	4.943
FCOS + CSPnonlocal [45]	86.10	5.404	5.817	3.980	3.708	4.332	4.416
FCOS + nonlocal [33]	86.10	5.422	6.139	4.521	10.059	4.411	4.423
FCOS + CBAM [15]	86.10	5.303	5.654	4.381	4.870	4.380	4.260
FCOS + CCAM [14]	86.90	4.632	4.491	4.025	3.641	4.035	3.517
FCOS + SA	87.70	5.067	5.248	4.273	4.418	4.305	4.016

**Table 18:** The comparison results of attention modules in parameters and GFLOPs.

Method	Parameters (M)	GFLOPs
FCOS [13]	32.118	19.764
FCOS + SE [36]	32.126 (+0.02%)	19.764 (+0%)
FCOS + CSPnonlocal [45]	32.284 (+0.52%)	19.782 (+0.09%)
FCOS + nonlocal [33]	33.302 (+3.69%)	19.882 (+0.60%)
FCOS + CBAM [15]	32.127 (+0.03%)	19.764 (+0%)
FCOS + CCAM [14]	34.154 (+6.34%)	19.964 (+1.01%)
FCOS + SA	32.253 (+0.42%)	19.778 (+0.07%)

**Table 19:** The results of GA and SA fusion method.

Method	Malposition Accuracy (%)	ETT-Carina		ETT Tip		Carina	
		Mean Err. (mm)	Err Std. (mm)	Mean Err. (mm)	Err Std. (mm)	Mean Err. (mm)	Err Std. (mm)
FCOS	86.10	5.335	7.831	4.254	5.427	4.659	7.497
FCOS + SA + GA	83.96	5.225	5.306	4.304	4.376	4.425	4.032
FCOS + GA || SA	87.17	5.492	6.583	4.956	9.549	4.164	4.158
FCOS + GA + SA	87.97	4.868	4.953	4.143	4.157	4.016	3.350

**Table 20:** The effect of fusing global modelling attention and scale attention.

Method	Malposition Accuracy (%)	ETT-Carina		ETT Tip		Carina	
		Mean Err. (mm)	Err Std. (mm)	Mean Err. (mm)	Err Std. (mm)	Mean Err. (mm)	Err Std. (mm)
FCOS	86.10	5.335	7.831	4.254	5.427	4.659	7.497
FCOS + nonlocal*2	OOM	OOM	OOM	OOM	OOM	OOM	OOM
FCOS + CSPnonlocal*2	85.56	5.800	8.991	4.391	5.543	4.703	7.512
FCOS + CCAM*2	86.10	4.855	4.988	4.020	4.037	4.301	3.835
FCOS + SA*2	87.17	5.643	6.820	4.432	5.021	4.422	5.387
FCOS + CSPnonlocal + SA	86.90	5.727	6.518	4.646	5.267	4.685	4.732
FCOS + CCAM + SA	87.97	4.868	4.953	4.143	4.157	4.016	3.350

**Table 21:** The results of employing mask branch into FCOS.

Method	Malposition Accuracy (%)	ETT-Carina		ETT Tip		Carina	
		Mean Err. (mm)	Err Std. (mm)	Mean Err. (mm)	Err Std. (mm)	Mean Err. (mm)	Err Std. (mm)
FCOS [13]	86.10	5.335	7.831	4.254	5.427	4.659	7.497
CTFA	87.97	4.868	4.953	4.143	4.157	4.016	3.350
Seg (All)	87.97	4.909	5.179	3.939	4.468	4.043	3.121
Seg (ETT)	89.04	5.486	7.682	4.398	6.754	4.521	4.244
Seg (ETT + Carina)	90.11	5.334	6.752	4.088	5.989	4.329	4.253
Seg (ETT + Carina) + Fusion	91.18	4.911	5.114	3.689	3.802	4.238	3.862

**Table 22:** The results of adopting mask prediction or not in the post-process algorithm.

Method	Malposition Accuracy (%)	ETT-Carina		ETT Tip		Carina	
		Mean Err. (mm)	Err Std. (mm)	Mean Err. (mm)	Err Std. (mm)	Mean Err. (mm)	Err Std. (mm)
w/mask	85.56	7.438	10.552	6.389	10.672	4.329	4.253
w/o mask	90.11	5.334	6.752	4.088	5.989	4.329	4.253

**Table 23:** The visualization results.

Good	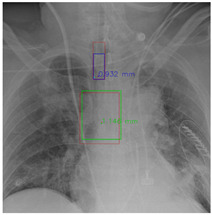	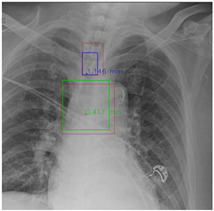
Medium	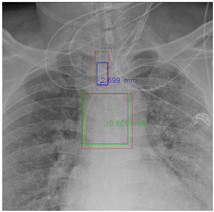	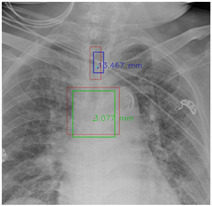
Worse	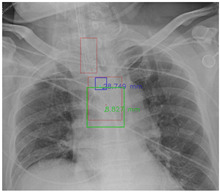	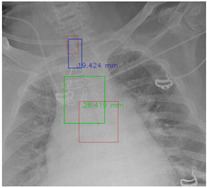

## Data Availability

The data that support the findings of this study are not publicly available due to the information that could compromise the privacy of research participants.
